# The Influence of Partner-Specific Memory Associations on Picture Naming: A Failure to Replicate Horton (2007)

**DOI:** 10.1371/journal.pone.0109035

**Published:** 2014-10-03

**Authors:** Sarah Brown-Schmidt, William S. Horton

**Affiliations:** 1 Department of Psychology and Beckman Institute for Advanced Science and Technology, University of Illinois at Urbana-Champaign, Champaign, Illinois, United States of America; 2 Department of Psychology, Northwestern University, Evanston, Illinois, United States of America; University of Melbourne, Australia

## Abstract

The results of two experiments by Horton (2007) show that speakers name a pictured object faster when in the presence of another person with whom the speaker has previously associated that object name. The first of those two experiments (Horton, 2007, Experiment 1) is the focus of the present research. This paper presents the results of three experiments designed to replicate and extend Horton's (2007) Experiment 1. The original findings were not replicated. These findings do not support the hypothesis that partner-specific memory associations facilitate object naming.

## Introduction

The use of language in conversation is shaped by knowledge of what information is and is not jointly shared by the conversational partners. During conversation, information that is mutually known between the conversational partners grows as conversational partners exchange information. This jointly shared information is known as *common ground* and is thought to provide the foundation for basic referential processes and communicative success [1]–[2].

A central question regarding the use of common ground is how this information is stored in memory. According to the classic view, personal common ground is encoded in memory through episodic, diary-like representations of joint experience [3]–[4]. More recent proposals alternatively suggest that individuals track one-bit cues to whether or not information is common ground [5], gradient representations of the degree to which information is common ground [6]–[7] or probabilistically combine privileged and common referential domains [8].

Beyond these questions about representation, another critical question is *how* individuals gain access to common-ground relevant information stored in memory. On the ordinary-memory view proposed by Horton and Gerrig [9]–[10], low-level associations that develop between individuals and information can afford sensitivity to common ground, without requiring access to full-blown representations of the circumstances that established common ground initially. With such associations in place, conversational partners can function as salient cues for the automatic retrieval of associated information from memory, through a resonance-like process similar to that described in domain-general models of recognition memory [11]–[12]. This process view is important because it suggests that, independent of the nature of the underlying representations (episodic, one-bit, or otherwise), access to suitable partner-specific representations may be able to occur routinely as a consequence of ordinary memory encoding and retrieval, obviating the need to presume special-purpose mechanisms. Indeed, a growing number of findings now highlight the fact that use of common ground in conversation is guided by basic memory processes [10], [13]–[15], emphasizing the need to understand the memory systems underlying this phenomenon.

One finding in support of the association-based view of common ground was a result reported by Horton (2007) [16]; this is the focus of the present research. Horton (2007, Experiment 1) created situations in which participants associated a term such as “banjo” with one partner, and then later named a series of pictures, including a picture of a banjo [16]. During the naming task the partner associated with the term “banjo”, or a partner not associated with that term, was seated next to the participant as they named the pictures. The results showed that speakers were 86 ms faster to name pictures when the person sitting next to them was associated with the picture (same-partner: 863 ms; different-partner: 949 ms). In addition, speakers were slower to name pictures with unfamiliar labels (pictures with unfamiliarized labels: 1088 ms). The 86 ms speed-up in naming times with familiar partners did not significantly correlate with individual subjects' ability to later recall with whom they had studied the pictures with. This lack of a correlation was argued to be consistent with the proposal that partners can serve as implicit memory cues.

The original goal of the present research was to replicate and extend the same-partner benefit in object naming, in order to examine the factors that modulate the use of association-based common ground. In what follows, we describe these efforts. The original experiment by Horton (2007) had 16 participants who each participated in three within-subjects conditions during object naming: same-partner (the object label was associated with the present partner), different-partner (the object label was associated with the other partner), and new pictures [16]. The estimated effect size for the critical partner effect (same vs. different) was *d* = 0.68. Based on this effect size, it should take 12 participants to reach 80% power and 42 participants to reach 99% power in our replication attempts (G*Power was used for all power calculations [17]).

## Experiment 1

Experiment 1 was designed to replicate Horton (2007, Experiment 1) [16], with materials developed by the first author, and a similar, but not identical experimental procedure compared to the original research. As in the original study, the goal of Experiment 1 was to evaluate the hypothesis that associations between partners and labels are sufficient to speed subsequent lexical access. As we describe above, the larger goal of this research was to first replicate Horton (2007, Experiment 1) [16], to set the stage for subsequent investigations of the mechanisms by which partner-associations guide perspective-taking in language use.

### Method

Experiments 1, 2a and 2b were approved by the University of Illinois at Urbana-Champaign Institutional Review Board. All participants provided written consent prior to participation.

#### Participants

Fourteen native English speaking participants from the student community at the University of Illinois participated in this experiment, in exchange for partial course credit or $8. This experiment was designed to run at approximately 80% power to detect the partner effect based on the results of Horton (2007) [16].

#### Materials and Procedure

At the beginning of the experiment, participants were introduced to two different research assistants (one male, one female) who served as partners during the task. Participants were informed that the partners would be switching in and out during the different phases of the experiment. As in Horton (2007) [16], the experiment occurred in three phases: Exemplar generation, Picture naming, Partner identification.

#### Exemplar Generation Task

During the first phase of the task, participants were seated in front of a computer screen. One of the two partners (Partner A) was seated next to the participant while Partner B waited outside. On each trial the computer provided a clue number, and the participant told this number to the experimenter, who read off the associated category clue from a worksheet, e.g., “a musical instrument”. The participant then clicked the mouse and the computer displayed the label for one category exemplar with spaces for some of the letters for five seconds, e.g., “B-NJ-”. During this time, the participant's task was to figure out what the word was (e.g., “BANJO”). After five seconds, the full word was displayed on the screen for five more seconds, at which point the trial ended. This phase of the task included 36 trials, including 16 target trials and 20 filler trials. After completing the 36 trials, Partners A and B switched places, and the participant completed the same task with the same category labels but with different exemplars. For example, the exemplar for Partner B for the category “a musical instrument” would have been “H-RP” (“HARP”).

Target stimulus labels were identical to those used in Horton (2007, Experiment 1) [16] (see Table 1). Filler stimuli were selected to be of a similar structure; like Horton (2007), they were selected based on category exemplar norms by Van Overschelde, Rawson, and Dunlosky [18]. The stimuli were organized into 36 distinct categories (16 target categories; 20 filler categories), with two exemplars per category (one associated with each partner). Stimulus presentation was controlled using Matlab's Psychophysics toolbox (PTB-3) [19].

**Table 1:** Target stimuli for Experiments 1, 2a, 2b.

Item	Category cue (phase 1)	Exemplar A	Exemplar B
1	an article of furniture	lamp	stool
2	a fruit	cherry	lemon
3	a weapon	arrow	rifle
4	a vegetable	onion	celery
5	a kitchen utensil	ladle	blender
6	a bird	ostrich	penguin
7	a four-footed animal	zebra	giraffe
8	an article of clothing	belt	gloves
9	an insect	grasshopper	caterpillar
10	a vehicle	motorcycle	helicopter
11	a type of ship or boat	yacht	canoe
12	a thing that women wear	lipstick	perfume
13	a type of footwear	socks	slippers
14	a gardener's tool	pitchfork	wheelbarrow
15	a carpenter's tool	pliers	wrench
16	a musical instrument	harp	banjo

#### Picture Naming Task

During the second phase of the task, participants were seated at the same computer and were asked to name a series of pictures as quickly as possible with its most commonly used label. Each trial began with a 500 ms fixation cross, followed by immediate presentation of the picture that was to be named. Once the participant named the picture, the experimenter (Partner A or B) hit a key to indicate whether the name was correct, after which the next trial began.

**Figure 1:**
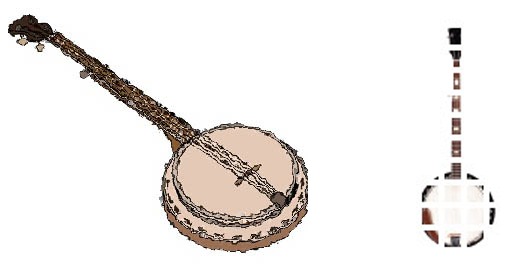
Example “banjo” stimulus from Experiment 1 (1a, left image) and Experiments 2a–2b (1b, right image).

Stimuli were of three types: Pictures with labels that were familiar from Phase 1 of the test, such as a banjo (n = 32); pictures with novel labels that were not studied in Phase 1 (n = 32), and unrelated fillers (n = 120). Labels for the familiar targets and novel pictures were identical to those used by Horton (2007) [16]. The 32 familiar targets represented 16 distinct semantic categories (two per category), and the 32 novel pictures were from 32 additional distinct categories. Unrelated fillers were selected to be similar to targets and novel controls without overlapping in meaning, though some fillers did fall within related semantic categories. As in Horton (2007) [16] the picture stimuli were degraded pictures of the critical image label. The original stimuli were photographs with a lattice of removed detail. By contrast, the pictures used in Experiment 1 were shaded drawings with a degrading filter that produced a wavy effect (see Figure 1).

Each participant completed half of the naming trials (16 target, 16 novel control, and 60 filler) with one partner and then the other half of the naming trials (16 target, 16 novel control, and 60 filler) with the other partner. The order of the two partners was randomized separately for each participant. In addition, the order of the stimuli and the assignment of critical stimuli to partner (e.g., whether the “banjo” would be named with Partner A or Partner B) was randomized separately for each participant. The partner who was participating with the participant sat next to them in the testing room and advanced the trials while the other partner sat behind a closed door in another room.

#### Partner Identification Task

During the third phase of the task, participants sat in the testing room alone while the two partners sat in the adjacent room with the door open. The participant viewed a series of words presented on the screen. For each test word, the participant answered the question “Is this one of the words from the guessing game?” by clicking on one of two response buttons (yes or no) at the bottom of the screen. If the participant responded “yes”, the computer asked the participant “Which partner was working with you in the guessing game when you had this word?”. The participant responded by clicking one of two buttons at the bottom of the screen that displayed the two partners' names (e.g., “Sally”, “John”).

Stimuli during the partner identification phase included all 32 target labels from Phase 1 that were subsequently named in Phase 2 (e.g., “banjo”), and all 40 of the filler labels from Phase 1. Each of these items should generate a “yes” judgment for having been studied during the guessing game. In addition, participants responded to labels for 40 of the 120 filler pictures from Phase 2 (which should be responded to with a “no” judgment since they did not appear in Phase 1), and 40 new words (which should also generate a “no” judgment).

### Analysis and Results

Data analysis focused on the latency to name the target pictures in Phase 2, and partner identification in Phase 3. All audio recordings were transcribed and word onsets identified by hand in Praat [20] by lab assistants who were blind to condition and unfamiliar with the research hypotheses. The primary independent variable was the nature of the partner-association (same or different) for experimental items in the picture naming task.

#### Picture naming

Naming accuracy was defined as participants using the anticipated name for the picture, and was high, 89% for target pictures and 90% for novel control pictures. Trials excluded from the analysis included cases where the recording was lost due to equipment failure (4% of the data), cases where the participant used the wrong label (10% of the data), and cases where the speaker used the right label, but was disfluent, e.g., “uh, banjo” (6% of the data). The remaining 721 trials (80% of all possible trials) were submitted to analysis. By convention, analyses are calculated separately using participant as the random variable (*t1*) and with item as the random variable (*t2*). For the analysis of the label familiarity effect (old vs. new labels), by-participant means were based on between 20–31 observations; by-item means were based on between 3–14 observations. For the analysis of the partner effect (same vs. different partner), by-participant means were based on between 6–15 observations; by-item means were based on between 1–10 observations. Average naming latencies for Experiments 1–3 are shown in Table 2.

**Table 2:** By-participant means by condition for Experiments 1, 2a–b (in milliseconds).

	Same-partner	Different-partner	Novel controls	Different - Same	New - Old
Experiment 1	846	849	964	3 ms (97)	117 ms (104)
Experiment 2a	1004	977	1247	−26 ms (217)	256 ms (241)
Experiment 2b	1023	1016	1241	−7 ms (138)	221 ms (176)

Mean differences are shown in the right-most columns along with the standard deviation of the difference (ms) in parenthesis.

The average latency to name target pictures was 117 ms faster than novel control pictures, an effect that was significant only by participants *t1*(13) = 4.22, *p*<.01; *t2*(61) = 1.74, *p* = .09. By contrast, participants were only 3 ms faster to name pictures that had previously been studied with the same partner in Phase 1 of the task, vs. a different partner, *t1*(13) = −0.12, *p* = .91; *t2*(30) = −0.86, *p* = .40 (note that the target stimulus “yacht” was dropped from the by-items analysis because it was named correctly only once). Supplemental analyses using mixed-effects models yielded the same pattern of results for all three experiments reported here. Whereas the effect of label familiarity was consistently observed (E1: *t* = 1.92, E2a: *t* = 3.51, E2b: *t* = 3.72), the effect of partner associations was absent (E1: *t* = *−*.76, E2a: *t* = .82, E2b: *t* = .54).

#### Partner Identification

Participants correctly responded that they had studied the label during Phase 1 of the task 94% of the time for filler labels from Phase 1, and 90% of the time for target labels from Phase 1. By contrast, incorrect “studied before” responses were only 7% for the labels associated with Phase 2 filler pictures, and 1% for novel labels. These high levels of accuracy show that even at the end of the task, participants had good memory for which items had been studied in Phase 1.

For all items that participants correctly indicated they had studied before, they further indicated which partner they had studied it with. Partner identification for Phase 1 filler labels was high—91% (Horton (2007) similarly reports partner identification rates of 85% for Phase 1 fillers [16]). We also note that for Phase 1 targets (which were subsequently named in Phase 2 with either the same or different partner), partner identification was also high, 80%. Partner identification rates for both types of stimuli were well above a chance level of 50% (single-sample *ps* <.0001).

#### Correlating explicit partner recall with Naming Times

Horton (2007) reported that the observed speed-up in naming times for same-partner trials was not significantly correlated with each participant's ability to explicitly recall the Phase 1 partner-label pairings for either the Phase-1 fillers or the Phase-1 targets [16]. While the present experiment was not designed to attempt to replicate these non-significant correlations, for completeness the correlations were computed. The difference in naming times between same-partner and different-partner trials was not significantly correlated with partner memory for Phase-1 filler labels (*r* = .37, *p* = .19), nor with partner memory for Phase-1 target labels (*r* = .50, *p* = .07).

### Conclusion

The results of this experiment are inconsistent with the findings of Horton (2007) [16]. We found no evidence that associations developed between individuals for a particular label such as “banjo” subsequently speed lexical access during spoken production of that label in the presence of the associated partner. By contrast, participants were significantly faster to name pictures with labels that were familiarized in Phase 1. Participants also showed good memory for the partner with whom they had studied the labels, suggesting that a failure to encode information about the partner during Phase 1 is not the source of the difference in findings.

Methodological differences between the original experiment and Experiment 1, however, may be in play. One difference between Horton's (2007) experimental design [16] and Experiment 1 was that participants completed Phase 1 and Phase 2 in the same testing room, whereas in Horton's experiment, participants moved to a different room for Phase 2. Additionally, the picture stimuli were different, and were noticeably *more* degraded in Horton's version of the task (Figure 1). The original motivation to use degraded stimuli was to emphasize partner effects (see Horton, 2007, p. 1121 [16]), thus the use of less degraded stimuli in the present Experiment 1 may have contributed to the lack of partner effects.

In what follows, we present the results of Experiments 2a–2b, which use the identical stimuli as Horton (2007, Experiment 1) [16], and a procedure more similar to the original study (e.g., participants completed Phases 1–2 in different rooms). Experiment 2a is a direct replication; Experiment 2b was a conceptual replication run at the same time.

## Experiment 2a

Experiment 2a was similar to Experiment 1; thus only differences in the experimental design are noted here. As before, our first step in the larger goal of investigating the role of partner-associations in perspective-taking was to replicate Horton (2007, Experiment 1) [16]. Given that the results of Experiment 1 were not consistent with Horton's experiment, Experiment 2a was designed as a sufficiently-powered direct replication of Horton (2007, Experiment 1) [16] using the identical materials and procedure as that study. Experiment 2b, which was run at the same time, was a conceptual replication designed in order to further the more general goal of understanding the factors that might modulate the use of association-based common ground.

### Method

#### Participants

Forty-nine native English-speaking participants from the student community at the University of Illinois participated in this experiment, in exchange for partial course credit or $8. This is three times the number of subjects run in Horton (2007, Experiment 1) [16], and should provide approximately 99% power to detect the partner effect based on the original findings. One additional participant was run but not included in the analysis due to a computer problem. No participant had participated in Experiment 1, and participants were randomly assigned to participate in Experiment 2a or 2b (see below).

#### Materials and Procedure

The basic procedure was identical to Experiment 1 with the exception that participants completed Phases 2 and 3 in a different room than Phase 1.

The materials, including all target and filler labels and all picture stimuli were identical to those used in Horton (2007, Experiment 1) [16]. The primary difference between the present Experiments 1 and 2a was that the picture stimuli in Phase 2 were more degraded in Experiment 2a (Figure 1). Additionally, rather than randomly assigning items to conditions for each participant, items were rotated across conditions through a series of experimental lists (list rotations were identical to Horton, 2007 [16]).

### Results

#### Picture naming

Naming accuracy was defined as participants using the anticipated name for the picture and was high, 92% for target pictures and 75% for novel control pictures. Trials excluded from the analysis included cases where the recording was lost due to equipment failure (0.2% of the data), cases where the participant used the wrong label for the picture (16% of the data), and cases where the speaker used the right label, but was disfluent (10% of the data). The remaining 2303 trials (73% of all possible trials) were submitted to analysis. For the analysis of the label familiarity effect, by-participant means were based on between 11–32 observations; by-item means were based on between 10–49 observations. For the analysis of the partner effect, by-participant means were based on between 7–18 observations; by-item means were based on between 17–26 observations. Average naming latencies for Experiment 2a are shown in Table 2.

The average latency to name target pictures was 256 ms faster than novel control pictures, *t1*(48) = 7.42, *p*<.0001; *t2*(48) = 3.50, *p*<.01, demonstrating a label familiarity effect. By contrast, participants were 26 ms *slower* to name pictures with labels that had previously been studied with the same partner in Phase 1 of the task, vs. a different partner; this difference was not significant, *t1*(48) = 0.85, *p* = .40; *t2*(31) = 0.94, *p* = .36.

#### Partner Identification

Participants correctly responded that they had studied the label during Phase 1 of the task 88% of the time for fillers from Phase 1, and 90% of the time for targets from Phase 1; by contrast incorrect “studied before” responses were only 9% for Phase 2 fillers, and 1% for novel labels. For all items with a correct “studied before” response, accurate identification of the Phase 1 partner for Phase 1 fillers was 84%, and for Phase 1 targets it was 77%. Partner identification rates for both types of stimuli were well above a chance level of 50% (single-sample *ps* <.0001).

#### Correlating explicit partner recall with Naming Times

As in Experiment 1, correlations between explicit partner identification and the difference in naming times between same-partner and different-partner trials were calculated. The correlation between the naming-time difference scores and partner memory for Phase-1 *targets* was not significant, *r* = .21, *p* = .15. Surprisingly, unlike Horton (2007) [16], the correlation between the naming-time difference scores and partner memory for Phase-1 *fillers* was significant, *r* = .42, *p*<.01. However, inspection of the data suggested that the latter correlation was largely driven by two outliers with naming time difference scores that were more than three standard deviations away from the by-participant mean. When these two datapoints were excluded, the correlation with filler memory was no longer significant, *r* = .26, *p* = .07 (the correlation with target memory was slightly larger in the reduced dataset, but still not significant, *r* = .27, *p* = .07). Given that this correlation is not significant when outliers are removed, and the correlation with partner memory for Phase-1 *targets* is not significant, it is unclear whether much can be concluded from this finding.

### Conclusions

Despite using the identical stimuli and procedure, and running three times as many participants as the original experiment, the results of Experiment 2a are inconsistent with those of Horton (2007) [16]. While we did observe a label familiarity effect on picture naming, and good memory for the Phase 1 partner, there was no evidence that partner associations facilitated picture naming. This failure to replicate suggests that the originally-reported finding, if real, may be of limited generalizability or of smaller magnitude than originally estimated.

## Experiment 2b

Experiment 2b was a conceptual replication of Horton (2007) [16], using task partners that were salient and distinct dolls, rather than human partners. Experiment 2b was run at the same time as Experiment 2a. The original goal was to determine whether the partner effect observed in Horton (2007) [16] was simply due to a salient memory cue or whether the knowledge that one's partner was also familiar with an object label was required to observe faster naming times in the same-partner condition. If the effect requires a sentient partner who genuinely experienced the familiar object labels, the logic was that the partner effect in Experiment 2b should not obtain. In addition, this experiment provides a test of the conceptually separate issue of whether a salient environmental cue could influence naming times. If a salient memory cue alone were enough, the highly distinct dolls in this experiment should be sufficient to yield the original partner effect.

### Method

#### Participants

Participants were 48 native English speaking participants from the student community at the University of Illinois, who participated in exchange for partial course credit or $8. No participant had participated in Experiments 1 or 2a, and participants were randomly assigned to participate in Experiment 2a or 2b. Five additional participants were run but not included in the analysis due to problems with the audio recordings.

#### Procedure

**Figure 2:**
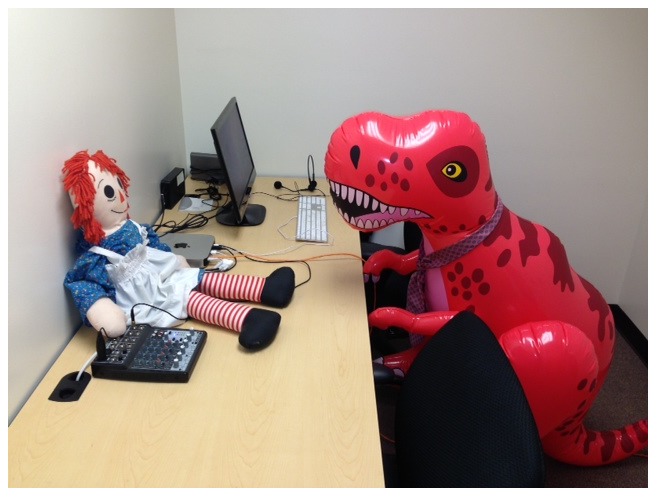
The dolls that served as context cues in Experiment 2b.

Experiment 2b was identical to Experiment 2a in every respect with the exception that rather than human partners, participants played the game with two different dolls, a red inflatable dinosaur, “Dr. Learnasaurus” and a Raggedy Anne doll, “Raggedy Anne”. The dolls were distinct in appearance and quite large; Dr. Learnasaurus was about 4′ tall and Raggedy Anne was about 2.5′ tall (Figure 2). A single research assistant swapped the dolls in and out of the rooms in the same way that the human partners switched in Experiment 2a. The research assistant also provided the category cues in Phase 1 and advanced the trials on the computer.

### Results

#### Picture naming

Naming accuracy was high, 90% for target pictures and 73% for novel control pictures. Trials excluded from the analysis included cases where the recording was lost due to equipment failure (0.2% of the data), cases where the participant used the wrong label (18% of the data), and cases where the speaker used the right label, but was disfluent (8% of the data). The remaining 2260 trials (74% of all possible trials) were submitted to analysis (Table 2). For the analysis of the label familiarity effect, by-participant means were based on between 5–32 observations; by-item means were based on between 5–47 observations. For the analysis of the partner effect, by-participant means were based on between 5–16 observations; by-item means were based on between 17–27 observations.

The average latency to name target pictures was 221 ms faster than novel control pictures, *t1*(47) = 8.67, *p*<.0001; *t2*(50) = 3.68, *p*<.001. By contrast, participants were 7 ms slower to name pictures that had previously been studied with the same doll in Phase 1 of the task, vs. a different doll; this difference was not significant, *t1*(47) = 0.36, *p* = .72; *t2*(31) = 0.6, *p* = .55.

#### Partner Identification

Participants correctly responded that they had studied the label during Phase 1 of the task 89% of the time for fillers from Phase 1, and 93% of the time for targets from Phase 1; by contrast incorrect “studied before” responses were only 6% for Phase 2 fillers, and 0.8% for novel labels.

For items with a “studied before” response, correct identification of the partner (doll) for Phase 1 filler labels was 78%; for Phase 1 targets participants correctly identified the Phase-1 partner 73% of the time. Partner identification rates for both types of stimuli were well above a chance level of 50% (single-sample *ps* <.0001).

#### Correlating explicit partner recall with Naming Times

The correlation between Phase-1 partner recall and the difference in naming times between the same-partner and different-partner conditions was calculated as before. The difference in naming times between same-partner and different-partner trials was not significantly correlated with partner memory for Phase-1 fillers (*r* = −.06, *p* = .71), nor was it correlated with partner memory for Phase-1 targets (*r* = −.04, *p* = .80); both correlations were notably smaller in magnitude than for the previous two experiments.

### Conclusions

This experiment was designed as a conceptual replication of the original study by Horton (2007) [16]; however, there was no evidence that associations between a highly salient contextual cue (in this case a large red dinosaur vs. a large doll) and specific labels speeded picture naming times.

## General Discussion

The results of two experiments fail to replicate the primary finding of Horton (2007, Experiment 1) [16], that participants are faster to name a picture when the individual sitting next to them is associated with the picture label. A third experiment that used highly salient dolls as cues also failed to observe a cueing effect on naming times. At the same time, participants in all three experiments were faster to name pictures with familiar vs. unfamiliar labels, suggesting the failure to replicate is not simply due to a lack of engagement. Participants were also significantly above chance at recalling the partner with whom they had studied each label, suggesting that memory for partners was intact.

### Implications for previous findings

The fact that none of the experiments observed a significant effect of partner associations on picture naming, despite being designed to run at high power, casts doubt on the notion that partner associations, at least as instantiated in this experimental paradigm, can reliably facilitate processes such as lexical access. In particular, Experiment 2a, which was designed as a direct replication, was run at 99% power, and provides strong evidence against the effect, if real, being as large as originally estimated.

In this context, it is important to note that the original study by Horton (2007) also reported a second experiment that was not the focus of the present research [16]. In this experiment (Horton, 2007; Experiment 2), during the exemplar generation task each of two partners was associated with object labels from distinct object categories (four different objects per category were used to establish each category-based association) [16]. The goal was to demonstrate that object naming could be facilitated on the basis of category-level associations with particular partners, a type of association that is undoubtedly present for many types of semantic categories. As reported in Horton (2007, Experiment 2), participants were 67 ms faster to name novel objects from categories associated with the current partner than objects from categories associated with the other partner [16]. This result conceptually replicated and extended the basic partner-specific priming effect, and appeared to indicate that partners did not have to be associated with specific lexical items in order to facilitate object naming. In light of the current findings, however, the status of this conclusion is less certain.

Experiment 2 from Horton (2007) involved a within-subjects design with 24 participants, and the effect size for the critical same-partner vs. different-partner comparison can be estimated at *d* = 0.43 [16]. Based on this effect size, it would take 74 participants to reach 95% power (and 103 participants to reach 99% power). In comparison, the effect of word-level associations found in Experiment 1 of Horton (2007) was estimated at *d* = 0.68 [16]. If forming lexical-based associations (e.g., Partner 1-> BANJO) is a pre-requisite to forming more general category based associations (e.g., Partner 1-> *Musical Instrument*), the effect of category-based associations on object naming reported in Horton (2007) Experiment 2 [16] may be no more reliable than the effect of word-level associations tested here. However, the present research was not designed to test this second effect, thus we leave open the possibility that the category-based effect reported in Horton (2007) Experiment 2 [16] may indeed be as large as was originally reported. In fact, it may be the case that accessing associations between partners and more general semantic categories may be a more robust process guided by distinct mechanisms, and as a result, more likely to consistently influence basic processes such as lexical access. Adjudicating these possibilities will require further research into category-based association effects.

### Implications for theories of common ground

In what follows, we discuss the implication of the present null findings for theories of common ground. As far back as Clark and Marshall's account of definite reference and mutual knowledge [4], it has been generally recognized that efficient and accurate attention to common ground during conversation must, in some fashion, rely upon access to appropriate memory structures. While the co-presence heuristics outlined by Clark and Marshall presumed explicit access to diary-like representations of information about what other individuals would likely know, the account proposed in Horton and Gerrig [9] focused on the potential role of domain-general processes of memory encoding and retrieval in shaping the information taken as common ground with others. In particular, this account described how other individuals could serve as salient cues for the automatic retrieval of relevant information from memory, via low-level priming mechanisms.

In the context of this memory-based account, Horton (2007) [16] was intended to demonstrate how this cue-based accessibility could function to facilitate lexical access during language production, even in the context of a non-communicative task such as picture naming. The idea was that, if suitably strong partner ∼ label memory associations were established during the initial encoding phase, then having that same partner as a context would facilitate subsequent production of those object labels, similar to other findings in which contextual cues “resonate” with representations in memory to increase the accessibility of associated information [11]–[12], [21]–[24].

The current findings, though, raise questions about the possible role that partner-specific memory associations might have in shaping speakers' access to relevant lexical information. In doing so, these results are reminiscent of other findings concerning the role of incidental environmental contexts, such as testing rooms and experimenters, on memory encoding and retrieval (see [25]). This body of research reveals inconsistent and/or minimal effects of environmental context on memory for, e.g., word lists, particularly in recognition or identification memory [26]–[28]. Some contextual-consistency effects have been found on free recall, as when participants are asked to imagine the studied item as integrated with the testing room [26], or when extreme contexts are used (e.g., on land vs. underwater) [29]. And while there exists some evidence of contextual-consistency effects on recall with relatively mundane room contexts [30] (see also the meta-analysis reported in [31]), a series of well-powered experiments failed to consistently observe this effect with experimenters and/or rooms as contexts [32]. Moreover, most of the positive findings in this domain have been reported for tasks involving explicit forms of memory. Environmental context effects on implicit memory tasks are even more elusive. For example, Parker, Gellalty, and Waterman [33] reported an influence of context reinstatement on a conceptual implicit memory task (category exemplar generation). However, subsequent work by Parker, Dagnall, and Coyle [34] led to the conclusion that those earlier results were due to contamination from explicit memory processes.

An open question, then, is whether partner-specific memory associations could, in fact, reliably speed lexical access in a different sort of experimental paradigm. In the present research, the lack of partner context effects may be related to the fact that the partner was incidental to the task (see discussion in [35]), along with the fact that the items between study and test were perceptually dissimilar (see [36]). By contrast, if item similarity from study to test were maximized, and partners made integral to the task, we might observe different findings. In real-world contexts, particular interlocutors are likely to be the focus of a nexus of information directly related to their social identities, their connection to you, and the conversational context more broadly. Establishing more clearly motivated partner-item associations could help increase the likelihood that the presence of a specific partner would reliably prompt retrieval of relevant knowledge. However, on a simple resonance-based memory mechanism, in which memory retrieval is automatic and cue-driven, the partner effect should not be directly mediated by communicative goals. Another consideration is that the test names were all fairly high-frequency nouns, which may be relatively insensitive to a single exposure in the context of a particular partner compared to low-frequency or even novel words which have few if any prior associations (see [37]). Likewise, a common task such as picture naming might be so overlearned as to be relatively insensitive to such contextual effects (even with the present use of degraded stimuli). More difficult or uncommon tasks may provide a better viewpoint from which to test for the existence of partner-specific associations on language use. Overall, then, similar to the broader context-dependent memory literature, pinning down these effects more definitively may require more systematic exploration of the circumstances under which particular partner-specific effects are most likely to obtain.

### Memory and Common Ground

Aside from issues related to the specific paradigm, there may be the temptation to view the current (null) results as reason to question broader claims about the need to better understand the basic memory processes involved in how speakers and listeners manage common ground more generally. We wish to emphasize, however, that there is a growing body of evidence that supports the notion that mechanisms of memory encoding and retrieval in conversational contexts play an important role in shaping language use. Much of this work is consistent with the claim that access to partner-specific memory representations can constrain language production and comprehension in ways relevant for common ground [6], [10], [14], [38]–[43]. Some researchers have questioned the need to assume partner-specificity, arguing instead that language processing can be guided by simpler memory representations in the form of “precedents” – that is, past experiences of referring to an object in a particular way, independent of a particular partner [44]–[46]. Although the issue of whether such precedents are partner-specific or not (or, alternatively, the circumstances under which they are more likely to be partner-specific) is a topic of active debate (see [40]), the common conclusion linking all of this work is that speakers and listeners will act in accordance with available memory representations. Although the present results provide no additional support for the hypothesis that partner-specific memory-associations facilitate object naming in a manner consistent with the predictions of the memory-based model of common ground, there is little doubt that sensitivity to common ground is based on representations in memory of joint experiences.

Additionally, recent work with memory-impaired patients suggests that multiple memory systems may be involved in the maintenance of common ground [47]. Episodic records of jointly experienced events featured prominently in Clark and Marshall's account of common ground and definite reference [4]. Patients with severe declarative memory impairment (including severe episodic memory impairment) show substantial impairment in the use of linguistic common ground, particularly when the information spans multiple utterances, suggesting that declarative memory plays a central role in certain aspects of common ground [48], (also see [49]). Importantly, the same patient population shows intact use of physical co-presence as a cue to common ground [48] as well as partner-specific accommodation of regional accents in speech perception [50] suggesting that non-declarative memory mechanisms may support other aspects of partner-specific language use. Understanding which types of memory representations are relevant to which types of partner-specific processes remains a central question for future research.

In conclusion, the present research fails to replicate the finding by Horton (2007, Experiment 1) that partner-specific associations influence object naming [16]. At the same time, it is undeniable that our previous experiences with specific individuals shape how we use language in a partner-specific manner. Moving forward, the ongoing development of models of conversation and common ground will continue to rely upon a better understanding of how fundamental aspects of memory shape the ways in which people interact.
